# Peer Support Among Nursing Students During Hospital-Based Clinical Placements: A Scoping Review

**DOI:** 10.1177/01939459261425304

**Published:** 2026-03-13

**Authors:** Marjolaine Dionne Merlin, Charles S. Bilodeau, Frances Gallagher, Émilie Léger

**Affiliations:** 1 School of Nursing, Université de Moncton, Moncton, NB, Canada; 2 School of Nursing, Faculty of Medicine and Health Sciences, Université de Sherbrooke, Sherbrooke, QC, Canada

**Keywords:** nursing student, peer group, peer support, peer counseling, education, internship, clinical placements

## Abstract

**Background:**

Clinical practice plays a crucial role in nursing education. Numerous studies have highlighted the positive impact of peer support during clinical placements. However, to date, no study has provided a comprehensive overview of the specific role of peer support within the context of clinical learning.

**Objective:**

We aimed to map literature describing the role of peer support among nursing students during hospital-based clinical placements.

**Methods:**

A scoping review was conducted. Five databases (CINAHL, PsycINFO, PubMed, ERIC, and ScienceDirect) were searched, yielding 550 articles. Two authors assessed eligibility using predefined criteria, extracted data from the selected studies, and performed a thematic analysis. The review is reported in accordance with the PRISMA-ScR guidelines.

**Results:**

Thirty-six articles were included. Peer support during hospital-based clinical placements emerged through 6 key roles: (1) reduces anxiety, (2) contributes to the feeling of not being alone, (3) facilitates adaptation to challenges, (4) contributes to the development of a sense of belonging, (5) promotes knowledge sharing, and (6) enriches the learning experience.

**Conclusions:**

The findings underscore the significant role of peer interactions in clinical learning, positioning peer support as a key component of nursing education. They highlight opportunities for educators to design and support collaborative learning environments, while also emphasizing the need for further research to inform the implementation and evaluation of structured peer support strategies in nursing education.

## Introduction

Clinical placements, where groups of nursing students learn in real health care settings, are a cornerstone of nursing education.^1^ These experiences allow students to bridge the gap between theoretical knowledge and practical application, fostering critical thinking and clinical judgment skills.^1,2^ Beyond technical learning, clinical placements also create opportunities for interpersonal interactions among students, which can influence their learning experience.^1,3,4^

One key concept in this context is peer support, defined as the emotional, informational, and practical support exchanged among students who share similar experiences.^2,3,5^ Numerous studies have stressed the positive impact of peer support during clinical experiences.^3-5^ Peer support can take many forms, including encouragement during stressful situations, sharing knowledge, and fostering a collaborative environment while learning.^3,4,6^ Nursing students develop a sense of belonging when they can share their experiences and challenges with peers who have faced similar situations in clinical settings.^7,8^ Receiving support from peers during clinical procedures contributes to enhancing students’ confidence.^9^ In clinical environments, where students often face high responsibility and performance pressure, peer support becomes particularly important for reducing anxiety and promoting a more positive learning experience.^5,6^ This aligns with social constructivist perspectives, which emphasize learning through social interaction and the co-construction of knowledge.^10^

Existing studies highlight the benefits of peer support, such as improved confidence, emotional well-being, and enhanced clinical performance.^3,4,6,9,11^ However, these findings are scattered across different contexts and focus on isolated aspects of peer interaction. To date, there is no comprehensive synthesis that examines the multifaceted roles of peer support, specifically within hospital-based clinical placements in nursing education. Addressing this gap is essential for understanding how peer relationships shape learning and for informing strategies that optimize clinical education.

Therefore, the purpose of this scoping review was to explore and synthesize existing literature on peer support among nursing students during hospital-based clinical placements. By clarifying its role and impacts, this review aimed to provide insights that can strengthen nursing education and better prepare students for professional practice.

## Methods

Given the current state of knowledge in the literature, a scoping review was selected to explore this concept in the context of hospital-based clinical placements. This methodological approach, as outlined by Arksey and O’Malley^12^ and by Peters et al,^13^ will adeptly facilitate the comprehensive mapping of pivotal concepts that form the foundation of this research area. The stakeholder consultations, as an optional step in the scoping process, will be conducted in a follow-up study to inform and validate our findings. We report this scoping review according to the guidelines outlined in the PRISMA-ScR checklist.^14^

### Step 1: Identifying the Research Question

The strategy proposed by Arksey and O’Malley^12^ and by Peters et al^13^ was used to develop our research question. The study focused on nursing students as the participants of interest. The primary concept examined was peer support within the context of hospital-based clinical placements. The research question guiding this scoping review was as follows: What is the role of peer support among nursing students during hospital-based clinical placements?

### Step 2: Identifying Relevant Studies

The scoping review focused on peer-reviewed research articles published in English or French. To capture recent trends in nursing education and peer support practices, we included studies published between 2000 and June 2024. This period was selected to capture the emergence and consolidation of peer support approaches in nursing education, which have developed in parallel with major pedagogical shifts in clinical education since the early 2000s, including the rise of collaborative and experiential learning models. A comprehensive search strategy was developed in collaboration with an academic librarian to ensure the optimal selection of keywords and the overall quality and reproducibility of the search process. Five electronic databases were systematically searched: CINAHL, PsycINFO, PubMed, ERIC, and ScienceDirect. The search strategy integrated both Medical Subject Headings (MeSH) and keyword combinations using Boolean operators (AND, OR), with search terms including but not limited to “nursing students,” “peer support,” and “clinical setting.” An example from one database can be found in Supplemental Appendix A.

Studies were included if they employed qualitative, quantitative, or mixed-method designs and specifically investigated peer interactions during hospital-based clinical placements. The review focused on research exploring how peer support contributes to nursing students’ academic development, clinical performance, emotional resilience, and professional identity formation within hospital-based clinical placements.

### Step 3: Selecting Pertinent Studies

**Figure 1.**
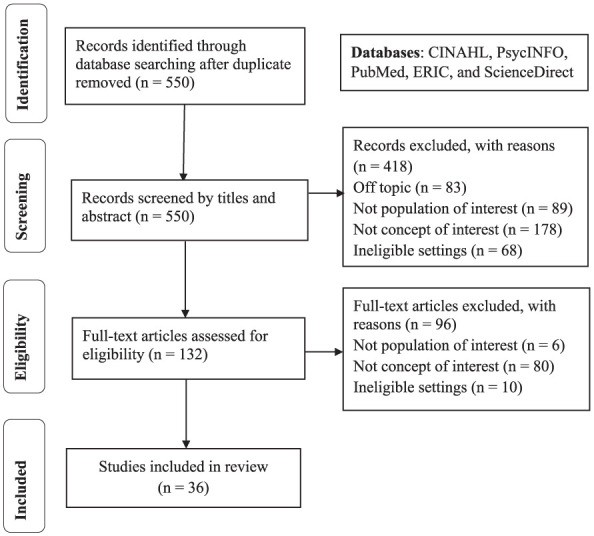
PRISMA flow diagram for the scoping review process.

The selection of articles followed a two-phase process, detailed in the PRISMA flow diagram presented in Figure 1. In the first phase, and throughout the entire process, at least 2 authors independently screened all titles and abstracts of the articles identified by the search strategy to ensure maximum rigor and transparency. A total of 132 articles met the inclusion criteria at this step. In the second phase, 2 reviewers independently conducted a full-text screening of the selected articles, leading to the exclusion of 96 studies that did not meet the inclusion criteria. Disagreements between reviewers were resolved through discussion and consensus. When consensus could not be reached, a third member of the research team was consulted to make the final decision. Articles were excluded, for instance, if they did not address peer support among nursing students or if they were set in nonclinical contexts such as classroom simulations or community-based programs. Literature reviews were only retained when they provided additional conceptual insights not available from individual studies. The final selection of 36 relevant articles was subsequently discussed and approved by all authors.

### Step 4: Charting the Data

A comprehensive data extraction sheet was utilized to gather significant data from the selected studies. We systematically charted key study characteristics, including authorship, year of publication, country of origin, research design, and stated objectives. Additionally, we extracted detailed information on the various roles attributed to peer support within hospital-based clinical education for nursing students. The first author was responsible for extracting all relevant data, while a second author independently verified the accuracy and completeness of the extracted data to ensure methodological rigor and transparency. Any discrepancies or uncertainties identified during the verification process were resolved using the same consensus-based approach described for the study selection phase, through regular discussions among the authors. Once consensus was reached, the final data entries were reviewed and approved by all members of the research team, reinforcing the reliability and credibility of the data extraction process.

### Step 5: Collating, Summarizing, and Reporting the Results

A key aspect of scoping reviews is the summarization of results in relation to the review question, as emphasized by Peters et al.^13^ This involves not only synthesizing the findings from the studies included but also contextualizing these results within the broader scope of our research question. By doing so, this step of the process provides a comprehensive overview of the existing literature, identifies gaps in knowledge, and features areas for future research.

The thematic analysis was conducted by the first author, following the methodology outlined by Miles et al,^15^ with a particular focus on identifying the roles and dimensions of peer support described in the selected articles. Themes were derived through an iterative coding process, starting with inductive generation of initial codes from the extracted data, followed by comparison across studies, and progressively grouped into higher order categories. The research team reviewed and discussed these codes collaboratively to ensure they accurately represented the meaning and nuances of the selected papers. Regular team discussions throughout the analytic process enhanced the credibility and validity of the findings, resulting in a thorough and consistent interpretation of the data. The analysis revealed multiple and interrelated roles played by peer support among nursing students during hospital-based clinical placements, shedding light on its contributions to learning, emotional well-being, social integration, and professional development for their future role.

This scoping review did not involve the collection of primary data from human participants. All data were obtained from publicly available sources and published literature. Therefore, ethics approval and informed consent were not required.

## Results

The results of our study allowed us to comprehensively elucidate the multifaceted roles of peer support among nursing students during hospital-based clinical placements, highlighting the benefits of these interactions within the learning process. Most studies were published after 2011 and originated mainly from Europe and the United States. As presented in Table 1, most employed qualitative designs.

**Table 1.** Characteristics of Research Articles.

Baseline characteristic	Full sample	
	n	%
Year of publication		
2000-2010	9	25
2011-2020	21	58
2021-2024	6	17
Origin of the publications		
Europe	12	34
United States	8	22
Canada	4	11
Australia	4	11
Other^a^	8	22
Study design		
Qualitative	21	58
Quantitative	6	17
Literature review	6	17
Mixed methods	3	8

a Asia, Iran, Taiwan, Turkey.

Characteristics of the clinical learning context included the duration of clinical experiences, the clinical settings, and the academic year of the program (Table 2). A notable contextual gap emerged: Few articles reported the length of clinical placements, and slightly less than half specified the health care settings where these experiences occurred. In more than half of the reviewed articles, clinical experiences involved combining different cohorts within the same placement setting. Although no formal quality appraisal was conducted, the included studies were generally relevant and demonstrated methodological rigor. Most studies applied clear inclusion criteria and appropriate data collection methods, with findings consistent with their objectives.

**Table 2.** Characteristics of the Clinical Learning Context.

Baseline characteristic	Full sample	
	n	%
Length of clinical experience		
2-4 weeks	7	19
5-8 weeks	5	14
>8 weeks	4	11
Unspecified	20	56
Clinical context		
Medical/surgical	12	33
Maternity/pediatric	2	6
Geriatric/rehabilitation	2	6
Psychiatry	1	3
Several clinical contexts	7	19
Unspecified	12	33
Program year		
First	4	11
Second	5	14
Fourth	3	8
Combined	19	53
Unspecified	5	14

Comprehensive details regarding the authors, study objectives, and the different roles of peer support identified in the included articles are presented in Supplemental Appendix B.

**Figure 2.**
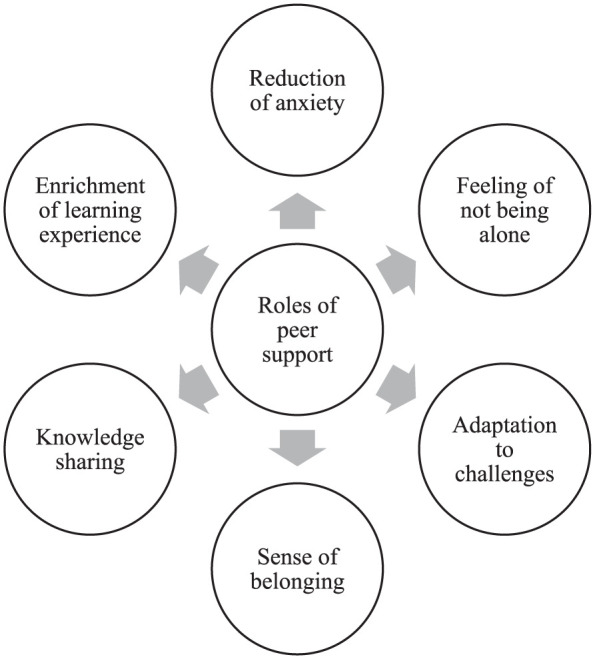
Multifaceted roles of peer support during hospital-based clinical placements.

The 6 themes emerged from a systematic, iterative coding process that involved identifying meaningful units, grouping similar codes into categories, and synthesizing these into broader themes. To ensure transparency and methodological rigor, this process was validated through collaborative review and consensus among the research team. These themes are interrelated and highlight the multifaceted roles of peer support within hospital-based clinical placements. Specifically, peer support: (1) reduces anxiety associated with the learning experience, (2) contributes to the feeling of not being alone in the experience, (3) facilitates adaptation to encountered challenges, (4) contributes to the development of a sense of belonging, (5) promotes knowledge sharing, and (6) enriches the learning experience. Figure 2 visually represents these roles of peer support identified in this review.

### Peer Support Reduces Anxiety Associated With the Learning Experience

Receiving support from peers significantly alleviates the anxiety commonly experienced during clinical placements, fostering a more positive and collaborative educational environment.^15-31^ It also plays a crucial role in moderating stress, which promotes a successful transition within the clinical learning environment for nursing students.^23,25^ Furthermore, this form of support enhances students’ capacity to navigate and succeed in new and challenging clinical settings.^25,27,32^ Overall, this form of peer support plays a pivotal role in the clinical learning environment, with significant implications for student well-being, academic persistence, and professional development.

### Peer Support Contributes to the Feeling of Not Being Alone in the Experience

The feeling of not being alone in the experience offers reassurance through close connections among students,^11,18,19,22,24,25,27,31-40^ creating a collaborative environment where concerns can be shared openly and encouragement readily offered. This dynamic enhances well-being and enriches the overall clinical experience. By providing both emotional and practical security^11,19,22,27^ and acting as a safety net during task execution,^24,32^ it offers reassurance and enables students to approach clinical responsibilities with greater ease.

### Peer Support Facilitates Adaptation to Encountered Challenges

Peer support plays a pivotal role in helping students adapt to the diverse challenges of clinical placements.^17-19,22,23,25,30,32-36,39-42^ By offering emotional reassurance that affirms students’ value and contributions,^17,18,19,23,25,32,39^ it strengthens their ability to develop effective coping strategies,^35^ build resilience,^25,35^ and sharpen problem-solving skills.^30^ Together, these elements foster competence and adaptability, enabling students to perform effectively in challenging clinical environments.

### Peer Support Contributes to the Development of a Sense of Belonging

Sense of belonging, defined as feeling part of the group of students, is fundamental to fostering inclusion and engagement within the clinical learning environment.^2,11,17-19,28,30,33-35,37,41,43,44^ By strengthening group cohesion,^2,37^ encouraging openness, and being grounded in mutual respect,^17,18^ it creates the conditions for effective collaboration and knowledge sharing. Support also fosters trust among peers, facilitates the formation of meaningful friendships,^2,17,19,28,30,37,41,43^ and uses humor to diffuse tension and overcome challenges.^35^ Together, these elements contribute to a positive learning climate that enhances clinical performance, personal growth, and overall academic success.

### Peer Support Promotes Knowledge Sharing

Support plays a crucial role in the sharing and building of knowledge, as mentioned in almost all the articles of this scoping review. It enables students to build on their peers’ knowledge through the exchange of clinical experiences and the reinforcement of learning through collaborative dialogue,^11,18,19,32,38^ while also allowing them to validate their own experiences and offer mutual guidance.^2,11,45^ By offering support during the execution of a task, the overall experience is enhanced, leading to improved outcomes.^45,46^ Additionally, receiving support fosters collaboration among peers, creating a more cohesive and productive environment where students can thrive and grow together.^32,47^

### Peer Support Enriches the Learning Experience

Peer support significantly enriches the learning experience,^11,18,20,23,32,34,35,37,38,40-44,46,48,49^ notably by enhancing students’ self-confidence,^16,17,19,24,26-28,30,31,33,39,47^ and by fostering critical reflection and professional development.^11,31,32^ This form of support not only bolsters the sense of self-efficacy^46^ and promotes autonomy,^28,47^ but also encourages leadership,^26^ engagement, and motivation.^28,38^ Furthermore, peer support helps students identify and connect with inspiring role models,^30^ strengthens collaborative skills, and enhances clinical reasoning abilities,^17,28,32,39,46^ thereby preparing them for complex decision-making in real-world clinical settings.

## Discussion

This scoping review described the role of peer support among nursing students during hospital-based clinical placements. Across 36 studies, 58% qualitative and conducted in diverse clinical contexts, the findings highlight the multifaceted contribution of peer support to clinical learning and student well-being. Peer support enhances the quality and depth of learning while offering psychological benefits and fostering professional development in a supportive learning environment.

Overall, peer support appears to operate through interconnected mechanisms. It nurtures psychological safety by helping students control emotional responses to clinical demands and enabling them to express concerns and seek help without fear of judgment. These conditions are consistently associated with lower stress and anxiety and sustained engagement in challenging situations.^5,16,17,23,25,27,50^ By articulating difficulties and hearing similar experiences from peers, students normalize their feelings, develop adaptive coping strategies, and maintain motivation throughout clinical learning. This process strengthens solidarity, mutual understanding, and resilience,^51^ helping to prevent negative learning experiences and reduce the risk of early withdrawal from nursing programs.^52^

Peer interactions also reinforce belonging and group cohesion, which correlate with initiative, openness to feedback, and persistence in learning.^2,11,17-19,33-35^ Students who feel accepted and supported by their peers are more likely to engage actively in learning opportunities, demonstrate initiative, and seek feedback from clinical educators, key drivers of deeper, more meaningful learning.

Peer support further contributes to professional development by promoting a collaborative learning environment. The diversity of perspectives within peer groups fosters richer discussions and a deeper understanding of clinical situations,^18,20,41,43^ while practical advice exchanged among peers often helps bridge gaps in knowledge and context. Beyond academic performance, peer support empowers students to recognize and seize growth opportunities, building confidence and competence.

Peer support among nursing students can be further interpreted through the lens of Kolb’s Experiential Learning Cycle,^53^ which conceptualizes learning as an iterative process involving 4 interrelated stages: concrete experience, reflective observation, abstract conceptualization, and active experimentation. Peer support facilitates progression through each stage of the learning cycle: (1) reduces anxiety to promote engagement in concrete experiences, (2) encourages reflective dialogue about clinical encounters, (3) enables cognitive reframing to support conceptualization, and (4) primes students for active experimentation through shared strategies and perspectives. Therefore, peer support simultaneously alleviates emotional barriers and strengthens engagement and confidence, reinforcing the cyclical nature of experiential learning.

Compared with prior reviews that broadly examine peer learning, this scoping review offers a focused synthesis of peer support within hospital-based clinical placements, an area still relatively underexplored. It outlines mechanisms through which peer relationships shape clinical learning and underscores the need to intentionally embed peer support into clinical education models.

### Limitations of the Present Study

Stakeholder consultations were not conducted as part of this review but are planned for a follow-up study to further inform and validate the findings. Although stakeholders’ perspectives were absent, 3 team members are nursing program educators, providing disciplinary insight during data interpretation. No formal quality appraisal was undertaken, which is consistent with the objectives of a scoping review. However, the included studies were generally relevant and methodologically sound. Several studies lacked contextual details, such as placement duration and health care setting, limiting transferability.

Study selection and data extraction were conducted in duplicate, but formal inter-rater reliability statistics (eg, Cohen’s kappa) were not calculated. Consistency was, instead, strengthened through iterative, collaborative discussions and consensus building. The evidence base is predominantly qualitative and does not allow for the establishment of causal relationships. Geographic concentration may limit generalizability, given variability in nursing education structures and peer support. Findings should therefore be interpreted with caution when applied to other contexts. Limiting inclusion to English and French language publications may have introduced language bias, potentially excluding relevant studies from regions where peer learning practices are emerging. This restriction could influence the comprehensiveness and cultural diversity of the evidence base. While gray literature was not included, the academic librarian’s expertise was invaluable in identifying and searching the appropriate databases.

### Implications for Nursing Education

These findings underscore the need to formalize peer support strategies within clinical education. Educators can implement structured peer-learning dyads to strengthen collaboration and reduce anxiety, guided peer-reflection activities to help students process clinical experiences, and clearly define peer-support roles during clinical orientation to foster belonging and confidence.^54,55^ Collaborative learning units and peer-assisted activities, such as case discussions, can promote shared problem-solving and critical thinking.^56^

Given the diversity of clinical placement settings, peer support strategies should be adapted to specialty areas and student level. For example, approaches used in acute care environments may differ from those implemented in rehabilitation settings. Models that prioritize peer support may substantially enhance professional growth and teamwork, contributing to a cohesive, supportive learning environment.^56^ However, it is important to acknowledge that peer support may present certain challenges, such as dependence on peers, uneven distribution of workload, or interpersonal conflicts.^11^ Recognizing these challenges is essential to implementing strategies that promote balance and constructive collaboration. Ultimately, strategies that reduce anxiety and foster belonging can help students build confidence and critical thinking, laying the foundation for safe, collaborative nursing practices.

### Future Research

Research should move beyond description to evaluate structured peer support interventions, such as formal peer mentoring or collaborative learning units, to determine their effectiveness and feasibility.^55,56^ This includes examining measurable outcomes such as academic achievement, clinical performance, student retention, and psychosocial well-being across diverse learning environments and student populations. Mixed-method designs and focus groups with students and educators can validate findings and capture multiple perspectives. Expanding the evidence base will help position peer support as a foundational component of effective clinical education.

## Conclusions

By synthesizing evidence on peer support during hospital-based clinical placements, this scoping review addresses a notable gap in the literature. Peer support is a strategic pedagogical resource that stabilizes emotional experiences, enhances learning through shared sense-making, and accelerates professional socialization. Implementing peer mentoring and collaborative learning units can strengthen resilience and teamwork, optimizing hospital-based clinical placements and prepare nursing students for the complexities of professional practice.

## Supplemental Material

sj-pdf-1-wjn-10.1177_01939459261425304 – Supplemental material for Peer Support Among Nursing Students During Hospital-Based Clinical Placements: A Scoping Review Supplemental material, sj-pdf-1-wjn-10.1177_01939459261425304 for Peer Support Among Nursing Students During Hospital-Based Clinical Placements: A Scoping Review by Marjolaine Dionne Merlin, Charles S. Bilodeau, Frances Gallagher and Émilie Léger in Western Journal of Nursing Research
