# Supplementary material for: Peer Support Among Nursing Students During Hospital-Based Clinical Placements: A Scoping Review
Source: West J Nurs Res. 2026 Mar 13;48(6):679–86. doi: 10.1177/01939459261425304 (PMC13168598; doi:10.1177/01939459261425304)
Supplement: sj-pdf-1-wjn-10.1177_01939459261425304 – Supplemental material for Peer Support Among Nursing Students During Hospital-Based Clinical Placements: A Scoping Review [file sj-pdf-1-wjn-10.1177_01939459261425304.pdf]

Appendix A. Databases

CINAHL, PsycINFO, PubMed, ERIC, and ScienceDirect

Example of the search strategy for CINAHL (Cumulative Index to Nursing and Allied Health Literature)

(MH "Students, Nursing" OR “nurs* student*”) AND ("peer support" OR "peer-support" OR MH "Peer Counseling" OR MH "Peer Group") AND (MH "Education, Clinical" OR MH "Internship and Residency" OR "clinical setting*" OR "clinical education" OR internship)

Boolean Operators: Combine terms using AND/OR to refine the search

Publication Date: Limit to articles published from January 2000 to June 2024

Language: English and French

Research article

No geographic limits

Appendix B. Overview of the roles of peer support during hospital-based clinical placements

Authors	Purpose	Reduces anxiety	Feeling of not being alone	Adaptation to challenges	Sense of belonging	Promotes knowledge sharing	Enriches the learning experience
Ädel, Löfmark, Pålsson, Mårtensson, Engström & Lindberg (2021)	To elucidate health-promoting and -impeding aspects of peer-learning by examining nursing students' descriptions of learning together as peers, and how this might interact with their health		X	X	X		X
Aghaei, Babamohamadi, Asgari & Dehghan-Nayeri (2021)	To explain the facilitating and inhibiting factors of nursing students' adjustment to the internship					X	
Austria, Baraki & Doig (2013)	To explore the student nurse and patient experiences of collaborative learning when peer dyads are used for clinical instruction in the hospital setting	X					X
Bourgeois, Drayton & Brown (2011)	To describe an innovative model of supportive clinical teaching and learning for undergraduate nursing students				X	X	
Carey, Chick, Kent & Latour (2018)	To explore the extent of learning development across different year groups of child health nursing students when engaging in peer-assisted learning (PAL); To identify if PAL provides opportunities for optimizing education in clinical practice; To identify the types	X		X	X	X	X

Authors	Purpose	Reduces anxiety	Feeling of not being alone	Adaptation to challenges	Sense of belonging	Promotes knowledge sharing	Enriches the learning experience
	of interactions that occurred as part of PAL in the clinical setting						
Chojecki, Lamarre, Buck, St-Sauveur, Eldaoud & Purden (2010)	To describe the perceptions of second year nursing students' and clinical instructors' experiences with peer learning while in an acute pediatric clinical setting	X	X	X	X	X	X
Christiansen & Bell (2010)	To explore the impact of a peer learning initiative developed to facilitate, purposefully, mutually supportive learning relationships between student nurses in the practice setting	X	X	X	X	X	X
Dionne Merlin, Lavoie & Gallagher (2020)	To explore the literature on elements of group dynamics that influence learning in small groups among undergraduate students		X		X	X	X
Edgecombe & Bowden (2009)	To highlight the value of factors and issues that shape, motivate, guide and determine the nature of phenomena experienced by nursing students	X				X	X
Fertelli (2019)	To determine the effects of students' assessment of nursing process through the peer assessment method on critical thinking and peer support in a clinical setting				X	X	X

Authors	Purpose	Reduces anxiety	Feeling of not being alone	Adaptation to challenges	Sense of belonging	Promotes knowledge sharing	Enriches the learning experience
George, DeCristofaro & Murphy (2020)	To survey all four levels of Bachelor of Science in Nursing (BSN) students in a rural southeastern university regarding self-efficacy and their concerns about clinical experiences	X					
Grealish, Lucas, Neill, McQuellin, Bacon & Trede (2013)	To determine the viability of the innovation by (1) developing a preliminary understanding of what students were learning and (2) exploring stakeholders' perceptions about student learning			X	X	X	X
Harvey, Carter-Snell & Kanikwu (2024)	To better understand undergraduate nursing students' experiences of using peer support after clinical related critical incidents	X	X	X			
Henshall, Davey, Merriman, Strumidlo, Serrant, Brett, Watson, Appleton, Malone & COV-ED Nurse Study Group (2023)	To explore how the COVID-19 pandemic impacted on the resilience levels of student nurses across the United Kingdom		X	X	X	X	X
Houghton (2014)	To understand how nursing students adapt to clinical practice and raise awareness of strategies that can be used to enhance their learning experiences	X		X		X	X

Authors	Purpose	Reduces anxiety	Feeling of not being alone	Adaptation to challenges	Sense of belonging	Promotes knowledge sharing	Enriches the learning experience
Jessee (2016)	To synthesize what is known about the influence of sociocultural factors in the acute-care CLE on prelicensure nursing students' perceptions of learning, for the purpose of identifying factors that when modified may promote improvement of clinical reasoning skill	X	X				X
Kaihlanen, Salminen, Flinkman & Haavisto (2019)	To describe newly graduated nurses' perceptions of a final clinical practicum that can facilitate the transition from nursing student to registered nurse			X		X	X
Kelly (2007)	To compare how second and third year nursing students view effective clinical teaching in diploma and baccalaureate programs						X
Liang, Wu, Hung, Wang & Peng (2019)	To develop and implement a resilience enhancement (RE)-based project for Taiwanese nursing students during their Last Mile practicum		X	X	X	X	X
Lopez, Yobas, Chow & Shorey (2018)	To understand how undergraduate nursing students perceive and experience their clinical placements and to identify the factors that helped them build resilience	X	X	X			

Authors	Purpose	Reduces anxiety	Feeling of not being alone	Adaptation to challenges	Sense of belonging	Promotes knowledge sharing	Enriches the learning experience
Mikkonen, Elo, Kuivila, Tuomikoski & Kääriäinen (2016)	To identify culturally and linguistically diverse healthcare students' experiences of learning in a clinical environment				X	X	X
Pålsson, Mårtensson, Swenne, Ädel & Engström (2017)	To investigate the effects of a peer learning model on nursing students' self-rated performance					X	X
Pålsson, Mårtensson, Swenne, Mogensen & Engström (2021)	To describe the collaboration between first-year nursing students using peer learning during their first clinical practice education		X	X		X	
Roberts (2009)	To explore the nature and value of peer learning for a group of pre-registration nursing students and aimed to enquire whether they learned from each other and if so, when and where this took place and to discover more about the processes used by the students whilst engaging in peer learning		X		X	X	X
Secomb (2008)	To provide a framework for peer teaching and learning in the clinical education of undergraduate health science students in clinical practice settings and make clear the positive and negative aspects of this teaching and learning strategy					X	X

Authors	Purpose	Reduces anxiety	Feeling of not being alone	Adaptation to challenges	Sense of belonging	Promotes knowledge sharing	Enriches the learning experience
Serçekuş & Başkale (2016)	To reveal students' thoughts about clinical environment and perceptions about the factors affecting clinical learning environments						X
Sprengel & Job (2004)	To discuss a peer mentoring project that was used to reduces anxiety during the students' first hospital experience	X					X
Stenberg & Carlson (2015)	To explore how student nurses' evaluated peer learning as an educational model during clinical practice in a hospital setting, and to compare perceptions among student nurses from year one and three	X	X			X	X
Taylor (2007)	To use self-reflexive means to explore critically a personal journey through collaborative work, between nurse teachers, students and mentors		X			X	X
Tornwall (2018)	To explore how academic nurse education is preparing student nurses to participate in a professional peer review process and to deal with the benefits and challenges of giving and receiving peer feedback	X			X		X
Van Horn & Freed (2008)	To describe students' clinical reflective processes as they worked individually		X	X		X	X

Authors	Purpose	Reduces anxiety	Feeling of not being alone	Adaptation to challenges	Sense of belonging	Promotes knowledge sharing	Enriches the learning experience
	and in pairs, solving problems while caring for patients						
Vuckovic, Karlsson & Sunnqvist (2019)	To investigate preceptors' and nursing students' experiences of peer learning in a psychiatric context during their clinical education	X	X	X		X	X
Walker & Verklan (2016)	To evaluate the effectiveness of a peer mentoring intervention on anxiety between groups	X					
Wang, Lin, Han, Huang, Hsiao & Chen (2021)	To explore the academic resilience of undergraduate nursing students during their adulting Nursing practicums and identify protective factors to mitigate their impact		X	X		X	X
Williamson, Kane, Plowright, Bunce, Clarke & Jamison (2020)	To investigate the views of student nurses and the placement staff about the similarities and differences in clinical placement experiences in Collaborative Learning in Practice (CLIP) areas compared to non-CLIP areas	X		X	X	X	X
Zentz, Kurtz & Alverson (2014)	To evaluate the effectiveness of peer-assisted learning in the clinical setting and to ascertain students' perceptions of fulfilling the roles of the professional nurse	X	X			X	X
